# A balancing act: exploring ethical and legal concerns associated with release of personal information in alert systems for missing persons with dementia

**DOI:** 10.1186/s12910-025-01214-4

**Published:** 2025-05-05

**Authors:** Adebusola Adekoya, Christine Daum, Antonio Miguel-Cruz, Lili Liu

**Affiliations:** 1 School of Public Health Sciences, Faculty of Health, University of Waterloo, Waterloo, ON Canada; 2 Department of Occupational Therapy, Faculty of Rehabilitation Medicine, University of Alberta, Edmonton, AB Canada

**Keywords:** Dementia, Missing persons, Alert system, Public disclosure, Ethics, Autonomy, Privacy, Stigma, Abuse

## Abstract

**Background:**

Technology, such as alert systems, can foster community engagement in locating missing persons with dementia and minimize potential harm. However, concerns arise about implications of public disclosure of missing individual’s personal information (such as age, photographs, physical descriptions, and medical conditions) within alert systems. Until now, there has been no review of these concerns, particularly in the Canadian context. Our study aimed to explore community members’ perspectives on the ethical and legal concerns associated with the release of personal information in alert systems for missing persons with dementia.

**Methods:**

Using a qualitative descriptive approach, we conducted semi-structured interviews with 18 participants: people living with dementia, care partners, service providers, first responders, and experts in ethics, policy, and the law from Canada and the United Kingdom. We conducted a thematic analysis of the interview data to inductively explore ethical and legal concerns.

**Results:**

Our findings identified the following concerns: *Balancing safety and privacy*,* stigmatization*,* risk of victimization and abuse*, and *informed consent*. There is a challenge of balancing safety with privacy due to the urgency of locating missing persons when sharing personal information publicly. Disclosure of personal information, such as cognitive impairment, can increase the risk of stigmatization, victimization, and abuse for both the missing individuals and their care partners. Unfortunately, conversations about alert systems and consent do not typically occur before someone goes missing, even though people living with dementia have the right to participate in these conversations.

**Conclusions:**

Alert systems can promote community involvement in locating missing persons with dementia but must balance safety and privacy concerns. Implementation of education and policies would mitigate stigmatization, victimization, and abuse. Early conversations with people living with dementia and their care partners to understand their preferences, along with an advance consent process, can help address consent concerns. Our framework, which emphasizes ethical and legal considerations, can guide policy, practice, and decision-making to support the autonomy of people living with dementia.

**Clinical trial number:**

Not applicable.

## Background

In 2020, over 55 million people worldwide were living with dementia—a number projected to nearly double every 20 years [1]. Dementia increases the risk of going missing by impairing wayfinding, making navigation and recognizing destinations difficult [2], putting individuals at risk of exposure to extreme temperatures, dehydration, and even death [3]. Between 40 and 60% of people living with dementia go missing at least once during the course of their disease and 5% will repeatedly become lost [4]. If these individuals are not found within 24 h, about 50% of them will sustain serious injuries or be deceased [5]. The global costs of dementia, estimated at $1.3 trillion in 2019, heavily strain care systems and individuals affected [6]. With no effective treatment available, dementia prevention and management, along with support for people living with dementia and their care partners remain a top public health priority [7].

Technology, such as alert systems, can engage the community in locating missing persons with dementia, providing extra eyes on the ground to support search and rescue efforts [3, 8]. An alert system uses wireless emergency networks, media outlets (e.g., radio or TV stations), electronic traffic signs, social media (e.g., Facebook and X, formerly Twitter), or apps to broadcast information (e.g., name, age, photo, physical descriptions, medical conditions) about missing persons with dementia at risks of harm [9]. Such alert systems are also in place for various populations, for example, the Green Alert for missing veterans [10], the Gold Alert for missing persons who are developmentally or cognitively impaired, and the Feather Alert for missing Indigenous persons in the United States [11]. Amber Alert for missing abducted children, utilized in many countries, including Canada, the United States, the Netherlands, the United Kingdom, the Czech Republic, and Poland [9, 12] are not appropriate for missing persons with dementia which do not generally involve a crime and occur much more frequently than missing children. Overuse could lead to public desensitization to alerts [9].

Alert systems such as Purple Alert (mobile app), developed by Alzheimer Scotland, allows family members to notify the community (app users) about missing persons with dementia [13]. Safeland, a mobile or web app, utilized in Sweden and some parts of the United Kingdom, enables community members to share information about emergencies, monitor their neighborhoods, such as keeping an eye out for missing persons, and reporting criminal activities [14]. Another type of alert systems commonly cited in the literature is the United States’ Silver Alert program implemented across various states and used by law enforcement to notify the public about missing persons with cognitive impairments [15].

The United States’ Silver Alert program is publicly funded in all but five states. However, reports on the success of the program in locating missing persons vary widely. Some studies reported the program helps locate missing persons with dementia [15–17], while others find it ineffective and question the community’s role in the process [18, 19]. Further, there are limitations to the program. Silver Alert policies vary across states and the criteria for issuing an alert may be based on factors such as age thresholds (e.g., 55, 60, or 65+), disability status, cognitive impairment, or being classified as “at risk” [15]. There are also concerns about alert fatigue as dementia-related missing incidents rise and media sensitivity declines [19].

Despite the limitations of Silver Alert, the program has garnered increasing attention in Canada as indicated by an online national petition to the Government of Canada for a National Silver Alert program [20]. Three Canadian provinces (Alberta, Manitoba, and Ontario) amended their Missing Persons Acts to incorporate “Silver Alert” or identify older adults in the Acts [21–23]. British Columbia has a citizen-led Silver Alert program for missing persons with dementia, cognitive impairment, and autism [24] and Quebec recently launched a Silver Alert pilot project for missing older adults [25].

Ethical and legal concerns exist about the potential harm from public disclosure of a missing person’s information in alert systems [19, 26]. Particularly, information about cognitive impairment or being classified as vulnerable may be used by scammers for criminal activity such as identity theft or financial fraud [27]. Alert systems can also compromise a person’s right to autonomy and privacy and how their personal information is disseminated to the public [15, 27, 28]. In Canada, the Privacy Act outlines rules for how government institutions collect, use, disclose, retain, and dispose individuals’ personal information [29], and the Personal Information Protection and Electronic Documents Act (PIPEDA) establishes the ground rules for how organizations engaged in a commercial activity collect, use or share personal information [30]. Privacy legislation could impact the implementation and use of alert systems, potentially causing delays in adopting the technology and releasing personal information. For example, information regarding missing persons shared by care partners must be vetted by police services before it can be released to the public [9].

Ethical and legal considerations are crucial in technology use for people living with dementia to protect their human rights [31]. While policies and criteria for alert systems provide guidance [32], they may not assist individuals or organizations to navigate the concerns associated with the release of personal information in alert systems. To date, there has been no review of ethical and legal concerns associated with the release of personal information in alert systems for missing persons with dementia, particularly in the Canadian context. To address this gap, we explored the perspectives of community members, including people with lived experience of dementia and expertise in ethics, policy, and the law on these concerns. We present a framework to guide policy, practice, and decision-making to support the rights of people living with dementia.

## Methods

### Design

This study utilized a qualitative descriptive design, which draws from the general principles of naturalistic inquiry to create an understanding of a phenomenon by exploring, analyzing, and interpreting the meanings participants ascribe to it [33, 34]. This approach is suitable when the goal of the study is to gain insights into a phenomenon that is not well understood, while also providing a comprehensive description of that phenomenon [33].

### Participants and recruitment

Participants included people living with mild dementia, care partners, first responders, service providers, and experts in ethics, policy, and the law regarding the use of alert systems to locate missing persons with dementia from Canada and Scotland. Purposive sampling was used to recruit participants with experiential or professional knowledge, aiming to capture a wide range of perspectives and in-depth insights [35]. We also used snowball sampling, a purposeful technique known as network sampling, where participants recommended others with similar interests and experiences [36]. Participants were recruited through our team’s professional networks (e.g., older adult and dementia advocacy organizations such as the International Consortium on Dementia and Wayfinding and AGE-WELL) by email and advertisements posted on websites and social media (e.g., X, LinkedIn). Inclusion criteria required that participants: (1) have experiential or professional knowledge on the topic of alert systems or ethical and legal concerns in the use of alert systems to locate missing persons with dementia; (2) were able to communicate (read and speak) in English. Exclusion criteria included not having adequate knowledge about the topic and not being able to articulate their perspectives due to moderate or severe cognitive impairments. A total of 18 participants met the inclusion criteria, agreed to participate, and none dropped out. The participants living with dementia who took part in this study had mild dementia and were able to provide informed consent independently. None of them required legal guardians or substitute decision-makers to provide consent on their behalf. For participants living with mild dementia, a teach-back method was used to determine their cognitive ability to engage in a one-on-one interview [37]. They were asked open-ended questions about the study information letter provided before the interview, including their understanding of the study procedure, risks, and what to do if they wish to withdraw from the study. The study received ethics clearance from the University of Waterloo Research Ethics Board (44447). No additional ethics clearance was required in the United Kingdom from the organization of the one participant (policymaker) in this study.

### Data collection and preparation

Data were collected between October 2022 and July 2023. Semi-structured interviews [38] were conducted with participants virtually (via Zoom) or in person at a mutually agreeable location by the researcher and participant. Eighteen individual interviews were conducted, and each participant was interviewed once. Individual interviews were approximately 38 to 75 min in length (average 57 min). An interview guide that contained demographic questions (e.g., age, sex, and ethnicity) and open-ended questions was used to guide discussion about participants’ perspectives on the topic (see Table 1 for interview questions). Definitions of ethical and legal concerns were provided during interviews as needed, and probes were used to elaborate on participants’ responses and clarify meanings. For example, participants were asked to give specific examples or additional information about their previous responses [39].

**Table 1:** Sample individual interview guide questions

1. From your perspective as a (person with dementia/care partner/service provider/first responder/expert in ethics, policy, or the law), what ethical concerns are associated with the release of personal information in alert systems? 2. From your perspective as a (person with dementia/care partner/service provider/first responder/expert in ethics, policy, or the law), what legal concerns associated with the release of personal information in alert systems? 3. What are the implications of releasing a missing person’s information to the public and as a part of the public record? a. In what ways can the release of personal information infringe on a person’s right to autonomy (freedom to make choices) and privacy? b. How does an alert system impact the person’s right to control when, how, and to what extent their personal information is released to others? c. How can the release of personal information to the public place a person with dementia at risk for harm? 4. In what ways may privacy outweigh safety and vice versa? a. How can persons with cognitive impairment or dementia retain their rights while using alert systems? 5. Is there anything else that you would like to contribute to today’s discussion that we haven’t addressed in this interview?	

Two group interviews, each with four to five participants (totalling nine), were conducted via Zoom to gather feedback on preliminary findings from the individual interviews. Participants received a summary of these findings by email beforehand. Six participants who could not attend the group interviews provided written feedback via Google Form or email, which was included in the final analysis. Three participants did not provide any feedback or respond to the group interview invitation. During the group interviews, individual interview findings were presented, and participants were asked if they agreed or disagreed with the findings and to offer additional comments as needed. Group interviews were approximately 49 to 70 min in length (average 60 min).

Individual and group interviews were all conducted by AA, a female registered nurse and doctoral candidate in Public Health Sciences with experience in qualitative research methods. Only the interviewer and participants were present during these interviews. Before starting the interviews, the researcher introduced herself, explained the study’s purpose and procedures, and obtained verbal or written informed consent from each participant. Observations made by the researcher during the interviews were documented in fieldnotes. All interviews were audio-video recorded and transcribed using technology-based transcription services. Transcripts were reviewed for accuracy. Participants were assigned a number to ensure anonymity and confidentiality.

### Data analysis

Data analysis occurred concurrently and iteratively with data collection. NVivo 12 was used to manage and organize the data. Thematic analysis was used to identify and analyze patterns within and across the data [40] relevant to participants’ perspectives on concerns associated with the release of personal information in alert systems. Initially, the first author (AA) listened to the recordings and reviewed the transcripts to become familiar with the data. This process aided in recognizing initial insights and understanding participants’ perspectives. Additionally, other team members (CD and AMC) also read the transcripts. Data were coded with descriptive words or phrases, generated inductively and reviewed multiple times to identify broader patterns (themes). Similar codes were refined, grouped, and organized into key themes, each supported by participant quotes. Analysis continued until saturation was reached. Team members (AA, CD, AMC) reviewed, discussed, and confirmed the themes (peer debriefing), refining them as necessary through two meetings.

Triangulation was achieved by collecting data from multiple sources, including individual and group interviews. Peer debriefing helped ensure the trustworthiness of our data and strengthened the study’s credibility [41]. Credibility was further enhanced through member checking, where participants validated the findings during group interviews and via a Google Form questionnaire [41]. Trustworthiness was also enhanced by prolonged engagement with the data, the inclusion of direct quotes from participants, and the researcher’s interpretation of the data [40].

## Results

### Participant characteristics

Eighteen participants (9 females, 9 males) from four Canadian provinces (Ontario, British Columbia, Saskatchewan, and Alberta) and Scotland, United Kingdom were interviewed. Three participants were people living with dementia, two were care partners, two were service providers, six were first responders (search and rescue and police), and five were experts in ethics, policy, and the law (lawyers, policymakers, bioethicist). Participant age groups ranged from 25 to 74 and half were between 45 and 54 years (mean age = 45-54years). Participants were predominantly White (*n* = 15), and the remainder were Filipino, person of mixed ethnicity, and Japanese (see Table 2).

**Table 2:** Participant demographics

Descriptive characteristics	n (%) (Total sample N = 18)
Age (years)	
25–34	1 (6)
35–44	1 (6)
45–54	9 (50)
>55	7 (39)
Sex	
Male	9 (50)
Female	9 (50)
Ethnicity	
White	15 (83)
Filipino	1 (6)
Japanese	1 (6)
Person of mixed origin	1 (6)
Role/Title	
Person living with dementia	3 (17)
Care partner	2 (11)
First responder (search and rescue, police)	6 (33)
Service provider	2 (11)
Policymaker	2 (11)
Lawyer	2 (11)
Bioethicist	1 (6)
Province/Country	
Canada	
Ontario	2 (11)
British Columbia	10 (56)
Saskatchewan	1 (6)
Alberta	4 (22)
United Kingdom	
Scotland	1 (6)

### Thematic findings

We describe the ethical and legal concerns associated with the release of personal information in alert systems under four key themes: (1) *Balancing safety and privacy*,* (2) stigmatization*,3) *risk of victimization and abuse*, and *4) informed consent.*

### Balancing safety and privacy

This theme underscores the challenge of balancing safety with privacy concerns when sharing personal information with the public and third parties. Participants emphasized the need to involve the public in locating missing persons with dementia to enhance safety while respecting privacy. Some noted the challenge of achieving this balance due to the urgency of locating the person, stressing the importance of considering the wishes of both the individual and their care partners. One participant living with dementia highlighted this challenge:

*It’s really difficult because there’s two different points here that are against each other. Because if they’re out and these alert systems can find them and bring them home. That’d be great. But then there’s the issue with privacy.* (P14)

For most participants, safety trumps privacy, as the primary goal of search and rescue is to locate the missing person quickly and alive. An experienced search and rescue manager of over 25 years, P13 expressed…“As a first responder, my first responder position would be, I want folks to be found sooner. So, I would lean on the side of safety over privacy.” Another participant, P5, living with dementia, who was at risk of going missing, emphasized the importance of safety and conveyed her willingness to share any necessary information to be found alive. She stated, “I would rather be found alive. So whatever information had to be passed on to help people find me that’s perfect.” Nevertheless, this emphasis on safety may have its drawbacks and come at a cost, as highlighted by a search and rescue member manager who had conducted many missing person searches:

*The benefits of revealing the information are greater than the cost. There’s a cost to releasing everything. Generally speaking*,* finding somebody alive*,* is going to outweigh having that information out there and they may feel violated*,* it’s a trade off. People do legally have the right to disappear.* (P9)

Participants had varied opinions on what information should be shared with the public through an alert system. Some worried about sharing medical information, while others felt it necessary to disclose critical information like a dementia diagnosis or required medications. Overall, participants agreed that sharing basic details—such as the person’s name, age, and physical descriptions—would help to identify them. A search and rescue member (P6) expressed: “It’s also an urgency concern. So, if they have dementia, if they might be making choices that are potentially not the correct ones, sharing that might be urgency in the eyes of the public.” A police officer (P16) with experience in missing person incidents and alert systems shared a contrary perspective by highlighting the need to share only minimal information.

*We are very careful and specific about what we release*,* and that it’s only to garner the assistance of the public with the least amount of personal information as possible*,* but enough for them to have the information they need to assist. We may speak in a generality that the person requires medication*,* we won’t say you’re diabetic*,* but you know*,* may require some medication to ensure their well being would be adding to the exigency.* (P16)

Safety is also related to the permanence of data in the public domain and the ways in which personal information is shared and utilized by third-party organizations. Participants voiced their concerns about the lack of control over how their personal data is shared and used by others, particularly online. A bioethicist (P17) specializing in gerontology expressed concerns about the public’s limited understanding of the government’s role in creating alert systems, the involvement of third-party organizations, and how these factors “collectively impact the person’s rights” in using such systems. Some participants also noted that information shared on the internet through an alert system cannot be completely deleted, as the internet does not “forget”. Additionally, concerns were raised regarding new Artificial Intelligence (AI) technologies, such as ChatGPT, analyzing information stored by third-party organizations. These concerns were elaborated by a lawyer who specializes in the rights of older adults at risk of harm and has extensive experience in policy development:

*We talk about the right to be forgotten but the reality is once things are on social media or for that matter*,* more broadly on the internet*,* we’re looking at new AI technologies where things may be scraped up. Particularly the point of being part of the public record on social media is the Forever aspect to it*,* I think*,* which is really very difficult. And the fact that there can be fakes*,* it could be spread and shared without synchronicity.* (P18)

### Stigmatization

This theme highlights stigmatization that could arise from being diagnosed with dementia, going missing, and using an alert system. Participants noted that society’s negative perceptions of people living with dementia, particularly those who go missing or whose diagnosis is publicly disclosed, can lead to feelings of shame and stigma. P6, an experienced search and rescue member expressed, “If you pull the dementia piece out of it, there’s a stigma with being a missing person. And to layer on top of that medical condition that is also stigmatized and not positively viewed makes it all the worse.” Due to stigma, care partners may hesitate to contact the police or seek public assistance when a relative goes missing. A care partner of a person living with dementia, who also specializes in dementia support, shared an example of the misconceptions and stigma associated with dementia:

*People don’t understand what dementia is. They don’t understand what causes it.*

*The word is terrible*,* like it comes from demented*,* which is demon*,* which is scary. People think that you did something to get dementia*,* or you must have done this…It’s such a feared and stigmatized disease.* (P15)

Many participants expressed that publicly disclosing a dementia diagnosis can lead to stigma and embarrassment for both individuals with dementia and their care partners, potentially affecting their reputation in the community. Concerns about stigma can also stem from cultural beliefs that view dementia as a private family matter, rather than something to be shared with others or publicly. A care partner whose father went missing and is an advocate of alert systems shared his concerns:

*Some family members are concerned about the stigma. They’re going to be judged*,* depending on the culture. It’s like we don’t want people to know that we’re dealing with this. So*,* they hide it. Are they concerned that we might be embarrassed or judged or look looked down upon as a family because you have this in your life?* (P3)

Some participants mentioned that care partners often experience guilt and stress when a relative goes missing, along with stigma from the public release of their relative’s dementia diagnosis. Those who feel “caregiver guilt” may blame themselves for their relative’s disappearance, feeling they failed to provide adequate care or ensure their safety—further reinforcing stigma. A service provider shared this concern, drawing from her experience supporting care partners of missing persons.

*I think sometimes for caregivers*,* it’s tough to have this all being managed*,* and they go missing for the first time. And is there shame about am I not looking after them properly? That’s my job*,* well*,* then I’m a terrible caregiver*,* like*,* is there any sort of perspective that they feel the community would see if the person went missing?* (P10)

Stigma and feelings of guilt can make care partners hesitant to disclose information about dementia to the police and search and rescue team when reporting a relative missing. This reluctance can delay public notifications and hinder the search process, according to an experienced search and rescue manager:

*Having done the interviews with people that have family members missing*,* it’s very stressful for them*,* because they’re looking at it from the point of view*,* are they to blame for the missing? So sometimes the information we’re getting is incomplete. You know*,* yes*,* they’re missing. And it’s even more stressful for the family*,* when we don’t find anybody.* (P8)

Participants also expressed concerns about how alert systems that utilize social media platforms like X and Facebook to share missing person’s information can lead to trolling and reinforce stigma for individuals living with dementia and their care partners. A bioethicist, P17, elaborated this concern: “There’s always a media backlash and social media harassment of family members. It’s not just the person with dementia, but the caregiver or anybody associated with the person that could also be subject to harassment and other negative impact.”

### Risk of victimization and abuse

Participants noted a perceived risk of victimization and harm associated with a dementia diagnosis, missing persons, and sharing personal information through an alert system. According to several participants, publicly disclosing medical information, like cognitive impairment, could heighten victimization risks and abuse for both to the individual and their care partners, even after the person is located. A service provider that offers dementia support to older adults, including missing persons with dementia, shared this concern:

*I think anybody with a cognitive impairment is exponentially at risk for those kinds of scams*,* identity theft and crimes and any release of information out there that identifies them as a person that is vulnerable. If a person with dementia is living in community and they go missing*,* and it’s out in the community within nefarious or people with not very good intentions now know that this person lives in the community and has dementia*,* then that puts him further at risk. And I do think that personal information*,* when it gets out into community*,* again add layers to what people know now about who’s living in their community*,* that would put them at further risk for being harmed.* (P10)

Interestingly, certain participants expressed that everyone, including those living with dementia, faces daily risks—such as using technology or maintaining a social media presence—and choosing to use an alert system is another risk to navigate. Disclosing excessive information, such as a dementia diagnosis or home address, could heighten the risk of victimization, making the individual a target for crimes like identity theft by scammers preying on susceptible older adults. A lawyer (P12) who also volunteers with search and rescue noted that knowing someone’s address and name could be used to “take advantage of that person.” This concern was further highlighted by a participant living with dementia who advocates for dementia support and alert systems:

*I think the biggest concern is that if you release the wrong kind of information*,* or too much information*,* you’re making someone who is already vulnerable*,* more vulnerable. If the wrong person sees it*,* and happens to find that person*,* they can use that information to defraud [them]. You don’t want to say*,* John lives here. And now all of a sudden*,* somebody zooms in on Google map to that.* (P2)

Participants highlighted that people living with dementia may face a heightened risk of abuse, which includes the misuse of their personal information because of their dementia diagnosis. An experienced first aid and search and rescue volunteer (P11) shared that information such as an individual’s last known location is made available to the public. He further stated that “people might take advantage of this information” by seeking out the person, knowing they have dementia. A policymaker, involved in implementing an alert system and providing support for missing persons and their care partners, voiced similar concerns about revealing the potential location of a missing individual:

*There are huge risks in sharing personal details [with] multiple people. I guess the legal concerns are almost doubled because we are talking about vulnerable people. You’re sharing very personal details*,* including geolocation and a huge amount of information about vulnerable person. So, the legal concerns are huge…the internet is a wonderful place, but it can also be misused.* (P4)

### Informed consent

This theme focuses on concerns surrounding informed consent in relation to decision-making about using an alert system, particularly regarding the sharing of personal information. Participants expressed that conversations about alert systems and consent typically do not occur before someone goes missing, even though people living with dementia have the right to be involved in these conversations. A participant living with dementia (P2) shared her perspective on this concern and a common misconception about the decision-making abilities of people living with dementia, “Too often, I’ve seen people with dementia treated like they no longer have any brains, like they can’t think for themselves or make decisions.” Another participant, a lawyer and search and rescue volunteer, clarified individuals’ rights concerning consent and the release of personal information in alert systems:

*Assuming that they’re competent*,* they have the same legal and human rights as a person who is completely competent. While a person is competent*,* they could actually give their consent to having information released an alert system*,* it becomes more complicated where they’ve never addressed the issue*,* or nobody’s ever asked the question.* (P12)

Other participants voiced concerns about the capacity of people living with dementia to make decisions and communicate their preferences for alert systems as their dementia progresses. They emphasized the importance of engaging people living with dementia early in conversations about the potential use of alert system and the public sharing of their personal information. A search and rescue member (P11) expressed, “Being aware that these things happen and having that conversation about what information the family is comfortable with us releasing or the individual releasing prior to their inability to give consent would be good.” A policymaker expanded on these concerns, emphasizing that the ability of a person living with dementia to make decisions can vary widely:

*The extent to which a person with dementia might be able to make those decisions would vary so much. It’s important that people should be involved in making decisions that affect them as much as possible to the greatest extent possible all the time. But it would seem to me that if a person is in the early stages*,* that’s a good time to have conversations with them about some of these difficult matters. And yet*,* again*,* to what extent are they able to make the decisions for themselves and express their wishes and concerns? That depends on that individual*,* their health situation and all kinds of other factors.* (P1)

Participants emphasized that care partners or substitute decision-makers (proxies) should be responsible for decisions regarding alert systems when a person with dementia is missing or unable to give consent themselves. However, proxy consent should still reflect the person’s preferences and wishes. A few participants also discussed the need for dementia support and how legal tools, such as advance care planning, could enable individuals to authorize the release of their personal information before they go missing, thereby enhancing their autonomy. A lawyer, however, raised concerns that people may not fully understand what they are consenting to:

*If you’re talking about advanced care planning which is the process for which a person explains their values*,* wishes and beliefs*,* and that can be then effectuated usually through a substitute decision making process. I guess you could say*,* if I’m wandering around*,* come and get me. And if you had it as part of advance care planning*,* it’s a peculiar thing*,* but like*,* what is it that you’re consenting to?* (P18).

## Discussion

We explored the perspectives of community members, including people with lived experience of dementia and expertise in ethics, policy, and the law on ethical and legal concerns associated with the release of personal information in alert systems for missing persons with dementia. These concerns were identified as *Balancing safety and privacy*,* stigmatization*,* risk of victimization and abuse*,* and informed consent*. Ethical and legal concerns overlap in areas such as privacy, consent, and public safety but differ in focus. Legal concerns emphasize compliance with privacy laws and data protection, while ethical concerns focus on balancing autonomy with the duty to protect vulnerable individuals. Both concerns must carefully navigate the public disclosure of personal information to ensure alert systems help locate missing persons without violating their rights or exposing them to further harm.

The literature presents the challenge of balancing safety and privacy concerns related to alert systems, as these two values are frequently seen as conflicting with one another [26, 28, 32]. While public disclosure of certain personal descriptors is necessary when seeking community assistance to safely locate a missing person, people living with dementia, like other adults, retain privacy, including the rights to go missing and control how, when, and to what extent their personal information is shared [27]. This raises complex questions about when safety should override privacy. Research has shown that care partners often prioritize the safety of their missing relatives over privacy rights [26, 32]. Likewise, our participants concurred that safety should take priority over privacy in urgent situations where a missing person with dementia faces a serious risk of harm or even death. The minimal use and disclosure of personal information is required in using alert systems [8, 28]. However, as our study identified, defining what qualifies as minimal necessary information poses a challenge. Moreover, the decision to publicly share medical information, such as a confirmed or suspected dementia diagnosis, in missing person cases remains debated [27].

A key finding of this study, not yet fully explored in the literature, is the concern about the permanence of data in the public domain and its potential use by third-party organizations. Privacy laws exist in many countries. For example, Canada’s Privacy Act [29] and Information Protection and Electronic Documents Act (PIPEDA) [30], the UK General Data Protection Regulation (UK GDPR) and the Data Protection Act [42], and the United States Privacy Act [43], regulate how personal information is collected, used, shared, and maintained by government institutions, commercial organizations, and federal agencies. These laws establish rules to protect individual privacy and ensure fair information practices. Personal information shared online or through social media can persist indefinitely, even if removed by law enforcement. Some participants expressed significant concerns about losing control over how their data is shared or stored by others. Recently, privacy risks have grown with new AI technologies like ChatGPT, as individuals’ data may not be adequately protected and could be shared with third-party organizations without their consent [44].

Our findings indicate that stigmatization is a significant concern in the release of personal information in alert systems; however, this concern has received minimal attention in the literature. Publicly disclosing personal information, such as dementia, can harm the reputation of people living with dementia and their care partners, perpetuate stigma and negative stereotypes about missing persons, ultimately influencing how they are perceived and treated by others in the community [27]. Similar to our findings, stigma may arise from cultural beliefs of dementia, for example, dementia might be seen as a curse or a consequence of wrongdoing, while changes in memory and behavior could be viewed as socially unacceptable [45]. Stigma can adversely affect an individual’s self-esteem and social inclusion, leading to distress, delay in seeking assistance, and a lower quality of life [45, 46]. The Canadian Charter of Rights for People with Dementia acknowledges the negative impact of stigma, particularly after disclosure of dementia, and aims to raise awareness of dementia and stigma as a human rights issue [47]. Although not legally enforceable, the Charter reinforces that people living with dementia have the same rights as all Canadians, challenging stereotypes that undermine their autonomy and ability to make decisions [47].

Family members may experience “caregiver guilt” when a relative goes missing, a feeling that can be compounded by the stigma of publicly disclosing personal information, such as a dementia diagnosis. While caregiver guilt is noted in dementia research, it has not been examined in relation to alert systems. Consistent with our findings, care partners can experience guilt due to the perceived moral obligation to care for and protect a relative living with dementia [48, 49]. This guilt can heighten caregiver burden and negatively impact mental and physical health, contributing to anxiety and depression, underscoring the need for targeted support and intervention [49].

There is a risk of victimization and abuse associated with the disclosure of personal information in alert systems. Our study findings indicate that people living with dementia might face a heightened risk of victimization and abuse, including the misuse of their personal data due to their dementia diagnosis. Similar to our findings, previous studies reported that information such as dementia diagnoses, home addresses, and photographs can increase the risk of identity theft and other crimes after the person is located [19, 26, 27]. The lack of clear, rigorous standards or policies regarding the minimal use of personal information can result in the potential misuse of such information within alert systems [8].

Concerns about informed consent focus on decisions surrounding the use of alert systems and the public disclosure of personal information. Informed consent requires a person to understand the purpose, risks, benefits, and alternatives options related to technology and to communicate a decision [50]. Policies and legislation on consent, especially in healthcare and personal information contexts, emphasize an informed, voluntary, and ongoing process, with guidelines for individuals lacking capacity [51–53]. The United Nations Convention on the Rights of Persons with Disabilities (CRPD) recognizes dementia as a cognitive disability and affirms that individuals retain their rights regardless of capacity [51]. In Canada, privacy laws require organizations to obtain consent from individuals or their substitute decision-maker for collecting, using, or disclosing personal information, with adults presumed capable unless proven otherwise [52, 53].

Although prior consent or consent from a proxy (substitute decision-maker) is required to release personal information [27], our study found that people living with dementia may not have been included in conversations about alert systems before going missing. This may stem from assumptions about their inability to make decisions and give consent [13, 15, 26]. Furthermore, proxy consent can be challenging when the person reporting someone missing or requesting an alert system is not the legal guardian [27]. As a result, our participants proposed the use of advance care planning—written instructions on important life and health care decisions—which can support a person’s autonomy and legal capacity when they are unable to make decisions for themselves [13]. However, people living with dementia and care partners may face barriers such as: limited awareness and understanding of the tool, the challenge of finding the appropriate timing for conversations, and inadequate support from health and social care professionals in making future health care decisions [54].

### Ethical and legal considerations framework for policy and practice

**Fig. 1:**
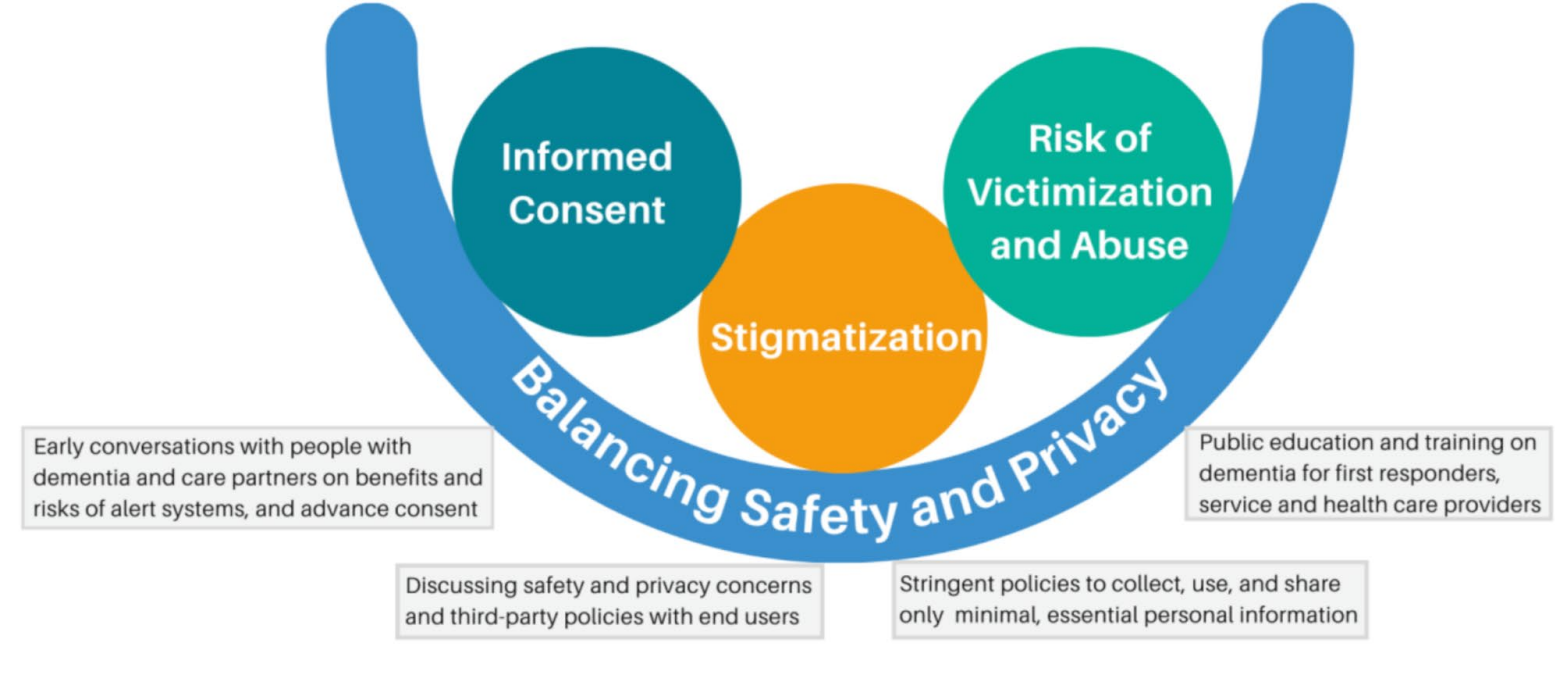
Ethical and legal considerations framework when releasing personal information in alert systems for missing persons with dementia

Our study highlights the need for ethical and legal considerations when releasing personal information in alert systems to support the human rights of people living with dementia. These considerations, including key themes identified in this study, are synthesized into a framework (Fig. 1) that can help inform policy, guide practice, and support decision-making regarding alert systems. While each theme can be considered individually, their interconnections are relevant. The colour scheme was intentionally chosen to highlight the uniqueness of each theme.

*Balancing safety and privacy*, depicted in blue, serves as a primary concern and acts as the overarching theme that integrates all other concerns, as the goal of sharing personal information is to engage the community in locating missing persons safely. This does not diminish the importance of other concerns but underscores the need to balance safety and privacy concerns. *Stigmatization*, represented in orange, can heighten the *risk of victimization and abuse* (depicted in green) due to the stigma surrounding dementia and going missing. Disclosing a dementia diagnosis may increase the risk of exploitation for people living with dementia. Concerns about *informed consent*, shown in teal, often stem from assumptions about the inability of people living with dementia to make informed decisions, which are also influenced by stigma.

The framework identifies considerations to address concerns related to sharing personal information within alert systems. Decisions regarding the release of personal information must strike a balance between the safety of people living with dementia and their right to privacy. While safety may take precedence in urgent cases of locating missing persons with dementia, it is important to recognize and address privacy concerns. It is vital to foster an ongoing and transparent dialogue about these concerns among people living with dementia, their care partners, and healthcare professionals or service providers. It is important for people living with dementia and their care partners to be aware of how their data will be used by third-party organizations and to understand privacy policies when agreeing to the use of alert systems. Additionally, law enforcement should promote the practice of deleting all data once the missing person is located, as this can help address concerns about data permanence and sharing [8].

To address concerns related to stigmatization, particularly society’s negative views toward people living with dementia and those who go missing, it would be prudent to implement public education and training for first responders, service providers, and healthcare professionals. This education and training should focus on dementia, the risks associated with going missing, and effective approaches for interacting with missing persons with dementia. Education can also help dispel cultural beliefs and misconceptions about dementia by engaging individuals with lived experiences and organizations such as Alzheimer Societies in community awareness campaigns [45]. Care partners can experience guilt and stress when their relatives go missing, making it essential for healthcare professionals and service providers to encourage strategies like self-forgiveness, support groups, and referrals to community services, including respite care, for these individuals.

Concerns about victimization and abuse resulting from releasing personal information in alert systems should be addressed through best practices that ensure stringent policies and safeguards. Alert systems should collect, use, and share only minimal, relevant information for locating the missing person. Personal information such as cognitive impairment should be disclosed, with consent, on a case-by-case basis and only when absolutely necessary to ensure the missing person’s safety.

Dementia-related missing incidents can lead to increased institutionalization and caregiver stress [3]. Individuals living in care settings, such as long-term care homes, may be at risk of getting lost and going missing if left unsupervised [55, 56]. Risk is often viewed negatively, portraying individuals with dementia as vulnerable, which can lead institutions to adopt risk-averse policies [57]. However, risk is inherent in daily life and should be assessed on a continuum, balancing safety with autonomy [58]. To mitigate the risk associated with going missing, discussions on repeat missing incidents, ongoing risk assessments [55, 57], and strategies such as alert systems should be integrated into admission procedures, consent forms, and care plans. A balanced approach should minimize harm while respecting individual preferences, values, and beliefs, as well as those of their care partners.

People living with dementia have rights to information and support to make informed care decisions [59]. Early involvement of people living dementia and their care partners in discussions about the risk of going missing, benefits and risks of data collection and sharing in alert systems, and their preferences can help address consent concerns. When consent is required from a proxy or substitute decision-maker, the person’s wishes and preferences should guide decisions. Consent should be regularly reassessed based on changing health needs and preferences. It is important to consider establishing a process for advance consent to release personal information and designate a substitute decision maker in case an alert system is required in the future.

While autonomy is a fundamental human right, people living with dementia, especially in care settings, may have diminished capacity in some areas but still retain decision-making ability, particularly regarding privacy [13]. However, healthcare professionals have a legal and ethical duty to prioritize safety, which may lead to privacy concerns being overlooked due to liability concerns [57]. Open discussions and collaboration among individuals, care partners, and healthcare professionals can help create personalized strategies that respect autonomy while ensuring safety [57]. Legal tools, such as advance care planning, as participants suggested, can facilitate this process and promote autonomy. Increasing education on advance care planning—from schools, colleges, and universities to primary care and service providers—could increase awareness and encourage proactive planning for individuals newly diagnosed with dementia and their care partners.

### Limitations

This study has limitations. A potential limitation of qualitative description is the lack of a required theoretical foundation. However, the researcher remained close to the data, prioritizing participants’ perspectives. Since this approach focuses on describing phenomena, it limits the ability to make broad generalizations [60]. The use of snowballing sampling allowed us to recruit participants with shared interests in the topic, higher health or research literacy and a greater inclination to participate, thus causing these characteristics to be overrepresented in the study. The majority of participants were White, which suggests that the perspectives of individuals from racialized groups outside of our networks might not have been fully captured.

## Conclusions

While there is strong public support for alert systems for missing persons with dementia, ethical and legal concerns around releasing personal information have been overlooked. Our study sheds light on these concerns through the perspectives of community members, including those affected. Public disclosure of personal information in alert systems can help engage the community in locating missing persons with dementia, enhancing their safety, but must be balanced with privacy rights. Disclosure of information such as cognitive impairment can contribute to stigmatization and increase the risk of victimization and abuse, highlighting the need for education, stringent policies, and safeguards. Early conversations with people living with dementia and their care partners are crucial to understand their preferences for sharing certain personal information. Advance care planning can also facilitate consent-related discussions. Our framework, which emphasizes ethical and legal considerations in information disclosure can help inform policy, guide practice, and enhance decision-making in alert systems to support the autonomy of people living with dementia.

## Data Availability

Data generated or analyzed during this study are included in this published article. To preserve the privacy and confidentiality of the study participants, audio-video records are not made available on open access.
